# A Review of Hypothalamic-Pituitary-Adrenal Axis Function in Chronic Fatigue Syndrome

**DOI:** 10.1155/2013/784520

**Published:** 2013-09-30

**Authors:** Cara Tomas, Julia Newton, Stuart Watson

**Affiliations:** ^1^Newcastle University, Newcastle upon Tyne NE1 7RU, UK; ^2^Wolfson Research Centre, Campus for Ageing and Vitality, Newcastle upon Tyne NE4 5PL, UK

## Abstract

Hypothalamic-pituitary-adrenal (HPA) axis dysfunction has been found in a high proportion of chronic fatigue syndrome (CFS) patients and includes enhanced corticosteroid-induced negative feedback, basal hypocortisolism, attenuated diurnal variation, and a reduced responsivity to challenge. A putative causal role for genetic profile, childhood trauma, and oxidative stress has been considered. In addition, the impact of gender is demonstrated by the increased frequency of HPA axis dysregulation in females. Despite the temporal relationship, it is not yet established whether the endocrine dysregulation is causal, consequent, or an epiphenomenon of the disorder. Nonetheless, given the interindividual variation in the effectiveness of existing biological and psychological treatments, the need for novel treatment strategies such as those which target the HPA axis is clear.

## 1. Introduction

Chronic fatigue syndrome (CFS) is a debilitating illness which was classified as a neurological disease in 1993 by the World Health Organisation (WHO) [1]. Symptoms of CFS include persistent fatigue, difficulty with memory and concentration, a disturbed sleep pattern, and severe muscular-skeletal pain [2]. Post exertional exacerbation of symptoms is common but not invariable [3]. The symptoms displayed vary markedly from patient to patient; some patients remain bedridden for very long periods of time, while others are able to manage their fatigue by staying within their own energy boundaries [4]. Diagnostic reliability is enhanced by the use of operational criteria such the Centre for Disease Control and Prevention Criteria [5], the Oxford Criteria [6] or the International Consensus Criteria [7, 8]. However, the heterogeneous symptom profile and absence of clear biological markers militate against confidence in the validity of CFS as a unitary diagnosis. It is not known, for instance, whether there is a core set of biological processes which underlie all cases of CFS or whether there are multiple processes (and if so, whether or not these potentially disparate processes converge on a final common pathway) [9]. 

**Figure 1:**
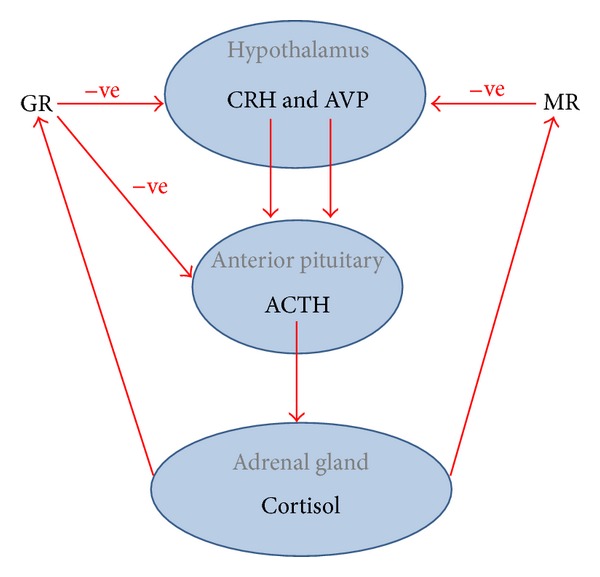
The effect of stress on the hypothalamic-pituitary-adrenal axis. The hypothalamus secretes both CRH and AVP from the paraventricular nucleus which stimulate the production of ACTH in the anterior pituitary. ACTH travels via the blood until it stimulates the adrenal gland to secrete cortisol. Cortisol acts via a negative feedback mechanism mediated by MR and GR in the hypothalamus and GR in the anterior pituitary, which ultimately causes a decrease in secretion of CRH, AVP, and ACTH. CRH: corticotropin releasing hormone; AVP: arginine vasopressin; ACTH: adrenocorticotropic hormone; MR: mineralocorticoid receptor; GR: glucocorticoid receptor.

Dysregulation of the biological systems which mediate the response to stress potentially has an important role in the aetiopathogenesis of CFS [1, 4, 10]. The neurobiological stress system comprises a range of networks that form intricate pathways; an important part of this is the hypothalamic-pituitary-adrenal (HPA) axis [11–14] which is a self-regulated feedback system which contributes to the maintenance of homeostasis and which is impacted by multiple factors such as time of day and physical and psychological stressors [2, 15]. There are a number of structures within the HPA axis, including the paraventricular nucleus (PVN) of the hypothalamus which releases corticotropin releasing hormone (CRH) and arginine vasopressin (AVP) which in turn stimulates the pituitary to secrete adrenocorticotropic hormone (ACTH) into the systemic circulation. The ACTH acts at the adrenal gland to stimulate the synthesis and secretion of cortisol. Cortisol is released in a pulsatile fashion and ensures strict regulation of both feedforward and feedback loops involving the HPA axis. Hence circulating cortisol activates mineralocorticoid and glucocorticoid receptors (MR and GR) and so decreases the secretion of CRH, AVP, and ACTH [16, 17]. This feedback mechanism is shown in Figure 1. Functional capacity of glucocorticoid receptors is considered, by some, to be the determining factor in the regulation of the HPA axis [18]. The effects of cortisol are both potent and extensive; it affects numerous physiological functions, for instance, in the regulation of the neuroendocrine and sympathetic nervous systems, modulation of the inflammatory response, inhibition of secretion of multiple hormones, and induction of lymphocyte apoptosis [19, 20].

## 2. Schematic of the Hypothalamic-Pituitary-Adrenal Axis Feedback Loops

See Figure 1.

## 3. Adrenal Steroid Metabolic Pathways

See Figure 2.

## 4. HPA Axis Function in Patients with CFS

Basal hypocortisolism was first reported in CFS patients in 1981 [21]. Cortisol concentrations have since been measured in blood, saliva, and urine in a number of studies with rather varying results (reviewed in [8]), but the notion of a hypocortisolaemic picture in CFS is supported by a meta-analysis [22]. Reduced cortisol levels are more apparent in female patients and also tend to occur during the later stages of the illness [22]. These abnormal cortisol concentrations may reflect differences in the biological mediation of the stress response or may be consequent on the differential nature/magnitude of the stressor engendered by the experimental procedure (e.g., the hospital visit or the venepuncture) in patients with CFS compared to comparator subjects [8, 16, 23]. Further, basal studies have also shown an attenuated diurnal variation [8, 24] particularly with a loss of the morning peak of ACTH [8, 20, 21, 25, 26] or cortisol [8, 20, 27] while challenge studies often, but not invariably, show a diminished HPA axis responsivity. This has been assessed using the ACTH, cortisol, and/or 11-deoxycortisol response to pharmacological challenge using, for example, dexamethasone combined with corticotropin-releasing hormone (CRH) [28], insulin [29], inflammatory cytokines, and metyrapone [30]; to psychological challenge (e.g., using the Trier Social Stress Test [31]), and to physiological challenge (such as wakening) [32–34].

The hypocortisolaemia, attenuated diurnal variation, and reduced responsivity to challenge seen in these cross-sectional studies may be mediated by upregulation of GR and MR, reduced hormone synthesis, or increased metabolism [8, 35]. The enhanced suppression of cortisol during the dexamethasone [36, 37] and prednisolone suppression test [38] supports the notion that increased functional activity of GR and possibly MR may have pathophysiological significance in CFS. However, as dexamethasone is metabolised via cortisol metabolic pathways, the enhanced cortisol suppression during the DST may therefore be caused not by GR upregulation but by reduced dexamethasone metabolism (as a consequence of the enzyme inhibition secondary to persistent hypocortisolaemia [39]).

**Figure 2:**
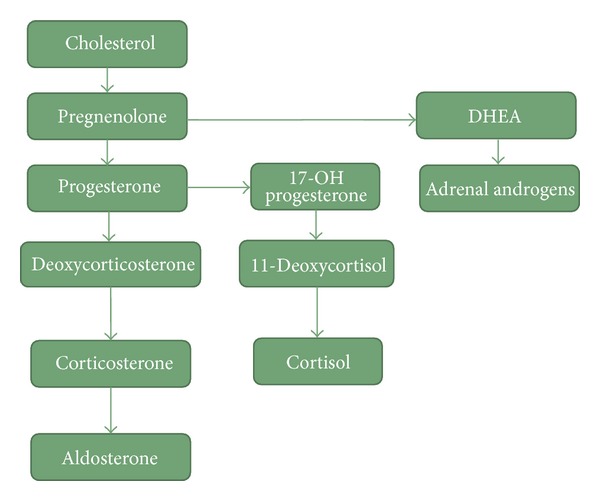
The metabolic pathway showing the steps involved in the synthesis of cortisol from cholesterol. Cholesterol is converted to pregnenolone by desmolase, which is then converted to progesterone by 3-B-hydroxysteroid. Progesterone is converted to 17-a-hydroxyprogesterone (17-OH progesterone) by 17-hydroxylase. The 17-OH progesterone is then converted to 11-deoxycortisol by the enzyme 21-hydroxylase. Finally, 11-deoxycortisol is converted into cortisol by 11-hydroxylase. DHEA: dehydroepiandrosterone.

The thesis that the hypocortisolaemia is caused by a shift in the balance of the various metabolic pathways (Figure 2) in steroid synthesis is tentatively supported but by no means proven by studies examining plasma concentrations of dehydroepiandrosterone (DHEA) and dehydroepiandrosterone sulphate (DHEAS) and by those which calculate the cortisol : DHEA ratio. DHEA levels have been shown to be normal [29], numerically [40] or significantly [41] increased; DHEAS levels have been reported to be reduced [42, 43], sand the cortisol : DHEA ratio has been shown to be normal [41, 44] or, in the larger study, reduced [40] in patients with CFS. 

## 5. Cause of the HPA Axis Dysregulation in CFS

It is valuable to explore the mechanisms which may explain the HPA axis dysregulation seen in adults with CFS. Specific genes (acting on the HPA axis or otherwise) which confer an increased risk of CFS have not been identified. There is, however, evidence of a heritable component to the disorder [45]. In addition, the role of early adversity also warrants consideration, particularly given the evidence of an increased rate of childhood trauma in patients with CFS. Around 50% of patients report at least one type of childhood trauma [46, 47]. It has been estimated that childhood trauma increases the risk of CFS between 6- and 8-fold [33, 48] with a graded relationship between the severity of the trauma and the risk of developing CFS [33, 46, 48]. Furthermore, an increased severity of symptoms has been noted in those who report childhood trauma [48]. It is increasingly established in other disease areas that childhood trauma acting via the HPA axis impacts the risk of disorder in adulthood, but it must also be remembered that early adversity is a broad concept which encompasses much more than childhood physical, emotional, and sexual abuse and neglect.

Animal models of early-life stress reveal HPA axis changes which persist into, or became evident in, adulthood [49]. There are a number of potential mechanisms. Variations in maternal care in rodents [50, 51], early life neonatal stress [52, 53], and childhood trauma in man [54–56] have been shown to increase methylation in the CpG-rich regions of a broad range of candidate gene promotors and transcriptional and intragenic sequences [57, 58]. One of the better studied has been the exon 1F promotor region of the GR gene (NR3C1). Increased methylation reduces transcription of the NR3C1 gene and so reduces hippocampal GR expression and increases glucocorticoid secretion in rodents [50, 51] and the cortisol stress response in man [52, 53]. Whilst this picture is not typical of HPA axis function in CFS patients, it nonetheless demonstrates the potential for neonatal and early childhood environment to impact on HPA axis function in adulthood. The methylation is measurable in blood lymphocytes and therefore provides a useful tool to examine the relationship between attachment and subsequent HPA axis function in patients with CFS [55]. FK506 binding protein 5 (FKBP5) impedes GR activation [59, 60]. Polymorphisms in the FKBP5 gene interact with early trauma to predict a number of stress-related psychiatric disorders. An elegant series of experiments by Elizabeth Binder's group suggests that this interaction is mediated by stress-induced methylation of FKBP5 during critical developmental windows [61].

Early life stress also influences brain development [33]. Teicher and colleagues [62] have recently demonstrated the sensitivity of the hippocampus, particularly the subiculum, to adversity. This is noteworthy because the subiculum has an important role in regulating the HPA axis; hence, neuroarchitectural change secondary to early adversity would be expected to alter the dynamics of the HPA axis in adulthood. Stress in adolescence or adulthood, such as that conferred by infection by microbes such as Epstein-Barr virus, *Coxiella burnetii*, and enteroviruses, is common but on occasion leads to CFS. It has been argued that this may be a result of disease in the central nervous system [63], but it is also worth considering whether preexisting HPA axis function or the nature or magnitude of the HPA axis response to this stressor plays a part in determining whether infection will precipitate CFS. We should be mindful that the adaptive response to the HPA axis changes of such genetic factors or stressors may confer an allostatic load which predisposes to CFS [64]. It has, for instance, been suggested that the allostatic response to persistent cortisol elevation may be a “switch” to hypocortisolaemia [35, 65–67] mediated by a relative increased reliance on AVP rather than CRH to drive [68], or on MR rather than GR to control, the axis [69, 70]. We are still a long way from understanding the evolutionary advantage of the altered regulation of the HPA axis in patients who have, and those who go on to develop, CFS; perhaps CFS is the cost of the cortisol response to challenge (including social challenge) which may be necessary to adapt to the complex dynamics of human social competition [71].

Another potential cause of disruption to HPA axis function is oxidative stress and a decrease in antioxidant capacity in addition to the presence of histone deacetylase (HDAC) [72]. Increased activity of HDAC 2 and 3 coincides with a decrease in plasma cortisol [14]. This theory has been cited as another possible cause of hypocortisolism found in patients. 

## 6. Impact of HPA Axis Dysregulation

Having argued that altered HPA axis function may have an aetiopathological role in CFS, it remains necessary to consider the link between cortisol and the typical symptoms of CFS. This may be mediated by immune mechanisms as a dysregulated HPA axis, particularly if characterised by hypocortisolaemia, has the potential to reduce the capacity with which HPA axis hormones can restrain the immune system. As a result, relatively minor physical or psychological stressors may be transduced into an inflammatory response by triggering the release of inflammasomes and subsequently proinflammatory cytokines [73, 74]. This process would be expected to evoke a pathological illness with symptoms such as those found in CFS patients [31, 75–77]. Further work is needed to quantify the inflammatory response in CFS patients. Cytokine-mediated inflammation may also explain the prevalent pain and hypersensitivity that affects CFS patients [78].

A vascular aetiology of CFS has also been proposed. This is a current research interest of our group and is exemplified by the relationship and overlap between CFS and postural orthostatic tachycardia syndrome (POTS) [79, 80] which typically presents with fatigue, dizziness, and an inability to exercise. HPA axis dysregulation, particularly hypocortisolaemia, can cause hypotension and may possibly mediate the fatigue experienced by CFS patients by inducing orthostatic hypotension and hence reducing cerebral perfusion [81].

## 7. Relationship between HPA Axis Dysfunction and Symptoms

The demonstrated association between the magnitude of HPA axis dysfunction and symptom severity highlights the relationship between the endocrinology and the disorder [8, 82, 83]. This is further emphasised by the demonstration that HPA axis dysregulation is a poor prognostic factor for CFS patients undergoing psychological treatment [22, 84]. The HPA axis dysregulation may have a causal role in CFS; it may be consequent on the disorder or it may be an epiphenomenon. Experimentally induced, or pathological, hypocortisolaemia (such as that seen in Addison's disease) is associated with symptoms typical of CFS, including fatigue, weakness, and abdominal pain, but it is also associated with a range of other features which are not typical of CFS [19, 85]. Further work to delineate the relationship between HPA axis dysfunction and fatigue in Addison's disease and other hypocortisolaemic states would be of benefit [86, 87]. 

Inactivity, sleep disturbance, psychiatric comorbidity, medication, and ongoing stress experienced by people with CFS will affect HPA axis function, and the findings that HPA axis dysregulation is more prominent in patients with a longer duration of illness suggest that the endocrine changes may be secondary [20, 88]. Interestingly, it has been proposed that these secondary endocrine changes may act to perpetuate the symptoms displayed by CFS patients [78]. The interindividual variation in HPA axis regulation in patients with CFS argues for a heterogeneous and multifactorial bidirectional relationship between the endocrine disturbance and the disorder [16].

## 8. Opportunities for Novel Therapeutic Strategies in Treatment of HPA Axis Dysfunction in CFS

Cognitive behavioural therapy (CBT) and graded exercise plans have demonstrated efficacy but with significant interindividual variation [4, 23, 78, 89]. These therapies modify illness perception and allow patients to make adjustment to optimise energy expenditure. CBT has been shown to increase cortisol levels by reversing some of the effects induced by low activity levels, depression and stress in early life [8, 82, 90, 91]. In addition, many pharmacologic treatments have been investigated for CFS including psychostimulants, corticosteroids, anti-inflammatories, and antidepressants [92–94]. There is currently no evidence to suggest that any of these medications have an advantage to patients though antidepressants are widely prescribed [95]. 

Low-dose hydrocortisone [96–98] and DHEA [42] have both been used as treatment agents in pilot studies in CFS, and have benefitted some patients. There is an argument for further trials of steroid treatment in patients selected on the basis of adrenal insufficiency, but the potential impact of long-term treatment including Cushing's syndrome, osteoporosis, extreme mood changes, and seizures cautions against this approach [99]. The HPA axis though remains a potential target for novel treatment strategies in CFS and this has been examined in studies utilizing animal models to examine traditional medicines with a putative HPA axis effect; for example, Shilajit, a traditional Indian medicine, reduced immobility and increased climbing behavior whilst increasing adrenal weight and corticosterone levels in the forced swim test in rats [72] and Myelophil, based on compounds used for fatigue in Chinese medicine, increased glucocorticoid receptor expression in the hypothalamus and hippocampus, and altered expression of cytokines such as interleukin (IL-10) and tumour necrosis factor-alpha (TNF-*α*) using the chronic cold stress and restraint model in mice [100].

One of the most interesting proposals is the switch to a new steady state from chronic hypocortisolaemia to a healthy, reactive state using the model-based predictive control (MPC) solution originally proposed by Gupta and colleagues [35]. This requires a pharmacologically induced short-term hypocortisolaemia in order to increase ACTH release to a threshold level following which a new equilibrium state is attained even in the absence of the pharmacological agent [19]. 

## 9. Conclusion and Further Research

HPA axis dysregulation appears to be associated with CFS. A credible body of evidence suggests a mechanism by which genetic and environmental factors (and their interaction) may serve to create an endocrine milieu which may impact on immune and vascular processes and thus putatively precipitate and maintain the symptoms experienced by those with a diagnosis of CFS. Nonetheless the abnormalities are subtle, and there is marked variation in basal and challenge tests in CFS patients and a real risk that these so-called abnormalities are simply confounds or epiphenomena. 

The findings that successful psychological treatments normalise the HPA axis dysregulation together with the reports that HPA axis dysregulation is a poor prognostic factor do give optimism that treatments targeting the HPA axis may have efficacy alone or in the augmentation of more established psychological, behavioural, or pharmacological treatments. 

The recent launch of the UK ME/CFS Research Collaborative [101] demonstrates the commitment of the government and associated funding bodies to pursue research into the understanding and treatment of this potentially debilitating disorder. This next decade may see an enhanced understanding of individual facets of CFS including its genetic and epigenetic signature, immune and vascular processes, the fine detail of HPA axis regulation, and the symptoms and psychological underpinning of the disorder. These should be examined using a network approach to map the intricate relationships and should allow consideration of whether CFS, as it is currently defined, is a unitary construct or if it represents multiple illnesses with different causes, albeit with similar symptom patterns. In addition, prospective studies may demonstrate vulnerability and trait factors and help to explain why some patients develop these symptoms. Hopefully, we can dispel any lingering Cartesian dualism and translate the psychological and biological understanding into holistic therapeutic programmes and novel treatment strategies. Progress is continuously being made; however, for patients who have had their lives destroyed, the development of a cure cannot come fast enough.
